# Optimal Rate Allocation in Cluster-Tree WSNs

**DOI:** 10.3390/s110403611

**Published:** 2011-03-25

**Authors:** Antoni Morell, Jose Lopez Vicario, Xavier Vilajosana, Ignasi Vilajosana, Gonzalo Seco-Granados

**Affiliations:** 1 Telecommunications and Systems Engineering Department, Universitat Autònoma de Barcelona, UAB Campus, 08193 Cerdanyola del Vallès, Spain; E-Mails: jose.vicario@uab.cat (J.L.V.); gonzalo.seco@uab.cat (G.S.-G.); 2 IT, Multimedia and Telecommunications Department, Universitat Oberta de Catalunya, Rambla Poblenou, 156, 08018 Barcelona, Spain; E-Mail: xvilajosana@uoc.edu; 3 World Sensing S.L., Baixada Gomis, 1, 08023 Barcelona, Spain; E-Mail: ivilajosana@worldsensing.com

**Keywords:** wireless sensor networks, contention free access, fair time slot allocation, distributed optimization, reduced signalling

## Abstract

In this paper, we propose a solution to the problem of guaranteed time slot allocation in cluster-tree WSNs. Our design uses the so-called Network Utility Maximization (NUM) approach as far as we aim to provide a fair distribution of the available resources. From the point of view of implementation, we extend here the authors’ proposed Coupled-Decompositions Method (CDM) in order to compute the NUM problem inside the cluster tree topology and we prove the optimality of this new extended version of the method. As a result, we obtain a distributed solution that reduces the total amount of signalling information in the network up to a factor of 500 with respect to the classical techniques, that is, primal and dual decomposition. This is possible because the CDM finds the optimal solution with a small number of iterations. Furthermore, when we compare our solution to the standard-proposed First Come First Serve (FCFS) policy, we realize that FCFS becomes pretty unfair as the traffic load in the network increases and thus, a fair allocation of resources can be considered whenever the price to pay in terms of signalling and computational complexity is controlled.

## 1. Introduction

Wireless Sensor Networks (WSN) have attracted the attention of the scientific community in the last years. This interest is mainly driven by the large amount of industrial applications that have appeared thanks to the cost reduction of the sensors. In this kind of networks, the standard IEEE 802.15.4 is usually adopted for the PHY and MAC layers [1,2]. This is because this standard has been specially designed with the aim of providing low cost devices with long battery life.

The IEEE 802.15.4 standard, in particular, offers two operating modes: the non-beacon mode and the beacon mode. In the first mode, nodes in the network are not synchronized and access the channel via unslotted Carrier Sense Multiple Access/Collision Avoidance (CSMA/CA). In the beacon-enabled mode, on the other hand, the network is synchronized by means of the beacons transmitted by a node acting as network coordinator and transmissions are organized in time slots. Apart from allowing the nodes to access the channel via contention (with slotted CSMA/CA in this case), this mode also provides guaranteed service to the nodes by allocating Guaranteed Time Slot (GTS) by means of a scheduling strategy. The adoption of GTS is included in order to assure QoS requirements in situations where CSMA/CA is less effective (e.g., under high traffic load). In that sense, new versions of the IEEE 802.15.4 standard such as IEEE 802.15.4e [3] or other coming standards such as WirelessHART [4] or ISA 100.11a [5] evolve with a clear definition of resources in order to manage the Quality of Service (QoS) of the ongoing connections. For example, IEEE 802.15.4e will be mostly based on GTS.

Concerning GTS management, the IEEE 802.15.4 standard follows a first-come-first-served (FCFS) strategy. The problem of such strategy is that the bandwidth allocation is not optimized, large delays can occur and fairness between users is not assured. In order to improve this strategy, several studies in the literature address the problem of bandwidth allocation and scheduling. The problem, however, is usually solved for star topologies (e.g., [6]) and further research is needed for the multi-hop case (mesh and tree-based networks). In [7], for instance, a review of scheduling strategies is presented but most of the algorithms are suboptimal and/or heuristic. Besides, the allocation problem for multi-hop networks presented in [7] is not efficiently solved and the studies referred in this work are related to generic wireless networks (instead of wireless sensor networks). Even in the case of the upcoming standards (IEEE802.15.4e, WirelessHART and ISA100.11a), where the use of guaranteed resources is emphasized, their allocation is still an open issue.

The main problem of multi-hop networks in the beacon-enabled mode is that this mode suffers from beacon collisions between different coordinators in the network. In a tree-deployed WSN, in particular, each parent in the network acts as a coordinator of its respective children. Therefore, each parent is in charge of its local synchronization by sending its own beacons and, then, collisions between the different beacon frames can appear if the system is not organized. In order to solve this issue, IEEE 802.15.4b Task Group presents several collision avoidance strategies [8]. Among all of them, it is of special relevance one strategy based on a time division approach as it is adopted by the Zigbee specification [9]. In particular, this approach consists in dividing time in such a way that the beacon frame of each coordinator is sent during the inactive periods of the rest of coordinators. It is worth noting, however, that the proposed techniques are focused on the beacon collision avoidance problem and that the slot allocation problem is not really solved. Indeed, some works in the literature depart from these strategies to introduce beacon frame scheduling techniques (see [10] for instance) but the scheduling of GTS slots inside each beacon frame is not directly addressed.

In this paper, we propose a resource allocation algorithm for distributing GTS slots in a tree-deployed wireless sensor network based on the IEEE 802.15.4 standard. Nevertheless, our approach is general and can be applied to other standards that define resources and provide means to share them among the sensors. In other words, although our results have considered the current version of the IEEE 802.15.4 standard, the contribution in this paper is not limited to it. More specifically, we derive a distributed algorithm that provides the optimal slot allocation based on a fair strategy that accounts for both the demand and the priorities of the different nodes. To do so, the problem is formulated as a Network Utility Maximization (NUM) problem, where the fairness among the different nodes can be adjusted, and the optimal solution is derived by means of a novel convex decomposition technique obtained by the authors [11,12]. In particular, this novel technique, referred to as the Coupled-Decompositions Method (CDM), has been extended here to the cluster-tree topology with the aim of efficiently addressing the distributed nature of the network. This extension raised new challenges, such as the computation of the primal and dual projections in a distributed manner or the convergence proof of the method. The main characteristics of the proposed approach are the following: (i) it is able to obtain the optimal solution in a distributed way (*i.e.*, the solution is obtained by distributing the optimization problem through the different nodes in the network), (ii) it converges with a significantly lower number of iterations than other state of the art techniques, and (iii) it requires a lower amount of signaling in the network. It is worth noting that the optimization problem is first formulated in a generic way in order not to restrict the solution to a given scenario. Indeed, the proposed algorithm can be easily extrapolated to perform the joint allocation of time and frequency, which is completely aligned with the philosophy of near future systems (e.g., IEEE 802.15.4e). After that, we focus on the beacon enabled mode of IEEE 802.15.4 with a cluster-tree topology and solve the GTS slot allocation problem by adapting the proposed strategy to this scenario. Our method is compared with the first-come-first-served (FCFS) strategy adopted by the standard, showing that our solution provides a significant improvement in terms of fairness performance as the traffic in the network grows.

The rest of the paper is distributed as follows. In Section 2, the resource allocation problem is formulated as a NUM problem and the proposed Coupled-Decompositions Method is described. After that, in Section 3, the proposed strategy is adapted to a realistic scenario based on IEEE 802.15.4 and numerical results are provided. Finally, the conclusions of the paper are presented in Section 5.

## 2. Distributed Fair Rate Allocation Algorithm

Let us assume a large-scale Wireless Sensor Network with *N* nodes that is monitoring a certain area. The mission of the network, as usual, is to gather the node measurements at the sink, where the information is processed. Let us assume that the nodes find their routes to the sink using best-effort solutions such as the Routing Protocol for Low Power and Lossy Networks (RPL) [13] or alternatives [14,15]. As a result, the initial graph of the network turns into a minimum spanning tree. Note that these techniques are very interesting in the context of WSNs since they keep the complexity of the routing protocol reduced, even given that they are suboptimal according to a certain performance metric. As important as routing is the Multiple Access Control (MAC) protocol, that is, how the sensors gain access to the wireless channel and how the available rate is finally distributed among the nodes. As discussed previously, two different families of approaches are being considered: (i) contention-based (e.g., CSMA/CA) and (ii) demand-based techniques (e.g., GTS allocation). The former is usually implemented due to its simplicity but it becomes inefficient in terms of throughput as the traffic load grows. The latter gives us the optimal solution once a global cost function is determined but it suffers from computational complexity and signalling requirements. In this paper, we propose a distributed algorithm for the second approach that converges fast to the optimal solution, which implies a reduced amount of signalling in the network. Furthermore, it is computationally lightweight.

**Figure 1.**
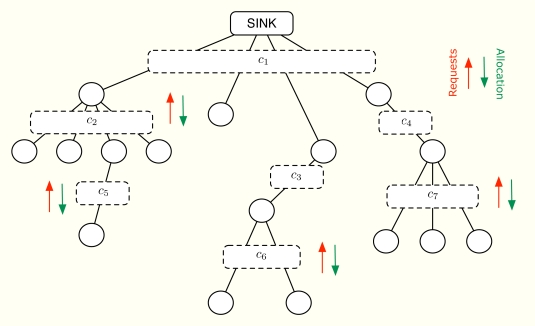
Demand-based multiple access. Rate grants are emitted and depend on the requests and on the rate capacities {*c_i_*} inside each cluster.

In the following, we assume that all the nodes in the network first make a rate request according to the amount of information they have to send. After running the distributed allocation algorithm, all of them get a certain transmission rate. The allocation depends on the rate requests, the priorities of the sensors, the minimum guaranteed rates and the maximum transmission rate at each level of the tree topology. All these parameters are known beforehand and the goal is to provide a fair rate distribution so that all the sensors in the network receive their transmission opportunities. On the contrary, note that a solution that maximizes the network throughput may prevent users with bad channel condition from transmitting. In practice, our solution is adequate for Frequency Division Multiple Access (FDMA) or Time Division Multiple Access (TDMA) since in both cases, it is possible to make an arbitrary division of the available total transmission rate in terms of bandwidth and time, respectively. Figure 1 provides a general view of our demand-based solution, where each dashed box represents one multiple-user communication channel between the parent node and its child nodes. Furthermore, a circle represents a node (sensor) and a line between two nodes simply indicates that they can communicate (using the corresponding multi-user channel).

### 2.1. Problem Formulation under Fairness Considerations

Let us consider the following optimization problem [16]

(1) $$\begin{array}{ccc} \underset{\left\{r_{j}\right\}}{\text{max}} & \sum_{j=1}^{N}U_{j}\left(r_{j}\right) & \\ s.t. & m_{j}\leq r_{j}\leq M_{j}, & j=1,\ldots ,N\\ & \text{Ar}≼\text{c} & \end{array}$$

with variables *r_j_* ∈ ℝ^+^ that represent the rate allocated to the j-th sensor. The functions *U_j_* : ℝ^+^ → ℝ are strictly concave, increasing and differentiable in {*r_j_* | *m_j_* ≤ *r_j_* ≤ *M_j_*}, where *M_j_* > *m_j_*, *m_j_* ∈ ℝ^+^. The vector ***c*** = [*c*_1_, . . ., *c_L_*]*^T^* contains the maximum transmission rate in the *L* channels of the network and the matrix ***A*** represents the network topology. Note that each wireless channel connects one parent node to its child nodes and that each entry *A*_*k*,*j*_ is either 1 if the flow from the j-th sensor crosses the k-th channel or 0 otherwise. Note also that Problem (1) defines a convex optimization problem [17,18]. Finally, we assume that there exists at least one strictly feasible solution to the problem, *i.e.*, a point that satisfies ***Ar*** ≺ ***c*** and *m_j_* < *r_j_* < *M_j_* ∀*j*, so that Slater’s constraint qualifications [18] (Section 5.2.3) are fulfilled and thus strong duality holds.

The optimization framework in Problem (1) is known to provide a fair distribution of resources if the utility functions *U_j_* are adequately chosen [19,20]. Moreover, we can specify the desired degree of fairness by fixing the parameter *γ* in the following family of utility functions [21] (Lemma 2) in Equation (2). Furthermore, *w_j_* ∈ ℝ^+^ is defined as the priority of the j-th flow and it is used to balance the flow allocation towards the j-th sensor once the value of *γ* is fixed (the higher the value of *w_j_*, the larger the allocation).

(2) $$U_{j}\left(r_{j};w_{j},\gamma \right)=\{\begin{array}{cc} w_{j}\text{log}\left(r_{j}\right), & \gamma =1\\ w_{j}\frac{r_{j}^{\left(1-\gamma \right)}}{1-\gamma }, & \gamma \neq 1 \end{array}$$

Roughly speaking, with *γ* → ∞ we get a max-min fair solution, that is, we do not allow to increase the rate of the i-th flow in the network if it implies (due to the constraints) decreasing the j-th flow given that initially *r_j_* < *r_i_*. That is the most stringent definition of fairness. The opposite situation is for *γ* = 0 and then the goal is to maximize the weighted network throughput. This solution is totally unfair since, depending on the network constraints, some sensors would have no transmission opportunities at all. An intermediate and well-established solution is obtained with *γ* = 1, a.k.a., proportional fairness. In this case, a small rate decrease in one flow is accepted if it implies a significant increase in other flows so that the network throughput is less penalized. For further details on fairness formulations, please refer to [21] and references therein. Note, however, that except for *γ* = 0, increasing the rate Δ*r* produces a bigger increase in utility when the sensor has a small rate allocation than when it is high.

In our WSN scenario, we assume that the network is built-up so that each sensor knows the route to the sink inside the cluster tree. Furthermore, each parent node has the information about the maximum transmission rate of its cluster, which is given by its own superframe configuration, and each sensor has evaluated its radio link quality, e.g., by means of the Packet Delivery Ratio (PDR). Given all this information, the goal is to compute a fair distribution of the available rate among the sensors. In the following, we focus our attention in the resolution of Problem (1) and we leave the practical implementation issues to Section 3. From the point of view of the optimization mechanism, note that one possibility is to gather all the requests at the sink node, compute there the optimal rate allocation and send it back to the sensors. Although there exist very efficient methods in the literature to solve generic convex problems, e.g., the so-called interior point methods [18] (Section 11.7), this approach is impractical in large-scale sensor networks as far as it implies an excess of signalling information. Therefore, there is a big interest in solving the problem as efficiently as possible, in a distributed manner. Efficient here means that: (i) the amount of signalling in the network and (ii) the computational load at the sensors must be kept small.

### 2.2. Existing Work

The problem we have formulated in Problem (1) is known in the literature as the Network Utility Maximization (NUM) problem [22] and several authors have employed classical decompositions, *i.e.*, primal decomposition [17] (Section 6.4.2) and dual decomposition [17] (Section 6.4.1) to solve it. Dual decomposition is usually preferred since it naturally provides a full distributed solution [23,24]. Indeed, the Active Queue Management (AQM) policy employed in the Transport Control Protocol (TCP) can be viewed as a convex optimization problem that is distributedly solved using a dual decomposition approach. Notwithstanding, optimally solving Problem (1) in a distributed manner is now time and signalling consuming due to the fact that the algorithms are based in a projected subgradient approach [17] (Section 2.3). In general, the convergence of the algorithms is slow and furthermore, it depends on a user-adjusted step-size, which is not necessarily optimally fixed.

In order to overcome these drawbacks, we have proposed the Coupled-Decompositions Method (CDM) [11,12]. It simultaneously intertwines primal and dual decomposition techniques in a single approach, converges much faster to the optimal solution and avoids the choice of a user-defined step-size. Note that there exist other primal-dual convex methods in the literature that are different to our approach. Some of them do not take into account the separability of the problem and thus are not true decomposition-based techniques [25,26]. Other works use primal and dual domains as well as the separability of the problem but they concatenate primal and dual domains in their algorithms instead of really mixing them [22,27,28], as we do in our CDM. In the following, we briefly review the classical decomposition methods. Afterwards, we extend the formulation of the CDM to rate allocation problems that exhibit a tree topology thus obtaining a novel distributed version of the method.

### 2.3. Review of Primal and Dual Decompositions

Let us consider the following equivalent version of Problem (1), which contains the additional variables ***y*** = [*y*_1_, . . ., *y_N_*]*^T^* with ***y*** ∈ ℝ*^N^*,

(3) $$\begin{array}{ccc} \underset{\left\{r_{j}\right\}}{\text{min}} & -\sum_{j=1}^{N}U_{j}\left(r_{j}\right) & \\ s.t. & m_{j}\leq r_{j}\leq M_{j}, & j=1,\ldots ,N\\ & r_{j}\leq y_{j}, & j=1,\ldots ,N\\ & \mathrm{Ay}≼c & \end{array}$$

Under this transformation, it is clear that given ***y***, Problem (3) can be separated into *N* independent problems, which are referred to as the primal subproblems in the specialized literature. In our particular case, these primal subproblems are

(4) $$p_{j}\left(y_{j}\right)=\{\begin{array}{cc} \underset{r_{j}}{\text{min}} & -U_{j}\left(r_{j}\right)\\ s.t. & m_{j}\leq r_{j}\leq M_{j}\\ & r_{j}\leq y_{j} \end{array}$$

Notwithstanding, we still need to find the optimal values in ***y***, that is, y^*^. Fortunately, it is known that the primal subproblems define a convex function on the variables *y_j_* and that the Lagrange dual variable *λ_j_* associated with the constraint *r_j_* ≤ *y_j_* is a subgradient [29] of *p_j_* at the point *y_j_* [17] (Section 5.4.4).

On the other hand, the primal master problem is defined as

(5) $$\begin{array}{cc} \underset{\left\{y_{j}\right\}}{\text{min}} & \sum_{j=1}^{N}p_{j}\left(y_{j}\right)\\ s.t. & \mathrm{Ay}≼c \end{array}$$

and the goal here is to find the optimal values of ***y***. Given that the subgradients of the subproblems are readily found once they are solved, the primal master problem can be computed using a projected gradient approach [17] (Section 2.3). In other words, ***s****_p_* = −[*λ*_1_(*y*_1_), . . ., *λ_N_* (*y_N_*)]*^T^* = − ***λ***(***y***) is a subgradient of $\sum_{j=1}^{N}p_{j}(y_{j})$ at ***y,*** where *λ_j_*(*y_j_*) is the value of the inner Lagrange multiplier of *p_j_* given the value of *y_j_*, and the following recursion converges to y^*^,

(6) $$y^{k+1}={\left[y^{k}-\alpha_{p}^{k}s_{p}^{k}\right]}^{†}={\left[y^{k}+\alpha_{p}^{k}\lambda^{k}\left(y^{k}\right)\right]}^{†}$$

where *k* indexes iterations, $\alpha_{p}^{k}$ is the user-defined step-size in primal decomposition and [·]^†^ is the projection to the set {***y*** ∈ ℝ*^N^* | ***Ay*** ≼ ***c***}.

Dual decomposition is the dual-domain version of primal decomposition and, in this occasion, we focus on the dual problem [18] (Section 5.2). In particular, we relax only the coupling constraint ***Ay*** ≼ ***c*** in Problem (3) thus defining the following dual master problem,

(7) $$\begin{array}{cc} \underset{\mu }{\text{max}} & \sum_{j=1}^{N}d_{j}\left(\mu \right)-\mu^{T}c\\ s.t. & \mu ≽0 \end{array}$$

where *d_j_*(***μ***) are the dual subproblems, defined as

(8) $$d_{j}\left(\mu \right)=\{\begin{array}{cc} \underset{r_{j},y_{j}^{k}}{\text{min}} & -U_{j}\left(r_{j}\right)+{\left[A^{T}\right]}_{j}\mu \;y_{j}\\ s.t. & m_{j}\leq r_{j}\leq M_{j}\\ & r_{j}\leq y_{j} \end{array}$$

where [***A****^T^*]*_j_* stands for the j-th row of ***A****^T^*. As in primal decomposition, a subgradient of a dual subproblem is found at no cost once *d_j_*(***μ***) is computed [17] (Section 6.1) and under some non-restrictive conditions, that subgradient is in fact the gradient (see [17] (Section 6.1.1)). In our case, a subgradient is obtained as [30] $A\;\delta_{j}y_{j}^{*}(\mu )$, where $y_{j}^{*}(\mu )$ is the optimal value of *y_j_* given ***μ***. Therefore, a subgradient of the dual master problem at ***μ*** is ***s****_d_* = Ay^*^(***μ***) − ***c.*** Finally, the recursion

(9) $$\mu^{k+1}={\left[\mu^{k}+\alpha_{d}^{k}\left(Ay^{*,k}\left(\mu^{k}\right)-c\right)\right]}^{+},$$

where [*a*]^+^ = max{0, *a*} and $\alpha_{d}^{k}$ is the step-size in dual decomposition, leads to μ^*^.

Taking into account that we consider cluster-tree WSNs in this paper, both primal and dual decompositions can be used to deploy distributed flow optimization solutions. However, these techniques suffer from convergence speed and the need of adjusting $\alpha_{p}^{k}$ or $\alpha_{d}^{k}$, as discussed before. In the next subsection, we extend the authors’ proposed Coupled-Decompositions Method (CDM) [11,12] to provide a fully distributed solution in cluster-tree WSNs. Different to [11], we propose here a single stage algorithm where each iteration of the technique takes into account all the nodes. In [11], the proposed solution had several stages and in each stage, the nodes that belonged to the same network level were optimized thus involving several iterations. Therefore, the total number of iterations grew exponentially with the number of levels in the network. This drawback is overcome in the enhanced version of the method that we describe next.

### 2.4. Proposed Algorithm

**Figure 2.**
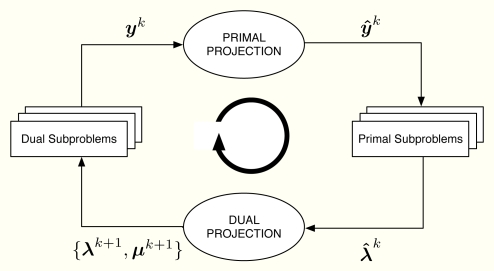
Block diagram of the CDM. The primal variables ***y****^k^*, ***ŷ****^k^* and the dual variables ***λ**^k^,* ***λ**^^k^*, ***μ****^k^* are exchanged among the building boxes of the proposed CDM.

Let us consider the problem formulation in Problem (3) and let us define *λ_j_* as the Lagrange multiplier (equivalently, dual variable) associated with the constraint *r_j_* ≤ *y_j_* for all *j*. As in dual decomposition, we keep ***μ*** = [*μ*_1_, . . ., *μ_L_*]*^T^* as the multipliers associated with the constraints in ***Ay*** ≼ ***c***. The technique we propose here intertwines primal and dual decompositions in a single approach and it is composed of four building blocks, namely: (i) dual subproblems; (ii) primal projection; (iii) primal subproblems and (iv) dual projection (see Figure 2). Before going to a more detailed description of the method, let us sketch out the four steps in the Figure 2, that form a complete iteration of the CDM, using a resource-price interpretation. From a practical point of view, this overview together with the contents of Section 2.5 shall suffice to develop our solution in real networks.

It is usual in convex optimization to think of primal variables as resources and dual variables as the prices to be paid for them. Let us assume that ***μ****^k^* fixes, at time instant *k*, the cost of transmitting one unit of rate through each of the available multiple-user channels. Note that the more congested the channel, the higher the cost is and that the cost for a non-congested channel is 0. Given ***μ****^k^*, each sensor obtains $\lambda_{j}^{k+1}$ as the sum of the prices in the multiple-user channels it uses. When the sensor computes the dual subproblem from $\lambda_{j}^{k}$, it decides how many resources to buy, *i.e.*, $y_{j}^{k}$, which essentially depends on its own utility function. However, the overall rate acquisition may violate the constraints in the network and it must be corrected. This is the task of the primal projection, that is, it gathers all the values in $y_{j}^{k}$ and modifies them to ${\hat{y}}_{j}^{k}$ in order to satisfy the network constraints. Thereafter, the primal subproblems estimate the price they will pay for the new allocation, *i.e.*, ${\hat{\lambda }}_{j}^{k}$. Finally, the dual projection looks at all the individual prices and tries to find a new consensus price ***μ***^*k*+1^. The entire process is repeated until all the sensors agree on the prices of the multiple-access channels.

Next, we describe one iteration of the method that starts with ***μ****^k^* and ends up with ***μ***^*k*+1^. Afterwards, we prove in Section 2.8 that ***μ****^k^* → μ*. In the following, a star as in y* or μ* indicates optimal values and a superindex *k* as in ***y****^k^* or ***μ****^k^* indexes iterations. Furthermore, we make use of some of the Karush-Kuhn-Tucker optimality conditions [18] (Section 5.5.3) of Problem (3). These are

(10) $$\lambda_{j}={\left[A^{T}\right]}_{j}\mu$$

(11) $$\mu_{j}\left({\left[A\right]}_{j}y-c_{j}\right)\;=0,\;j=1,\ldots ,L$$

(12) μ≽0

where [***A****^T^*]*_j_* is the j-th row of ***A****^T^*. Note that Equation (11) are the slackness conditions [18] (Section 5.5.2) related to the constraints ***Ay*** ≼ ***c*** in Problem (3).

#### 2.4.1. Dual Subproblems

From ***μ****^k^*, each sensor computes $\lambda_{j}^{k}={\left[A^{T}\right]}_{j}\mu^{k}$ as in Equation (10). Thereafter, the sensor solves the following optimization problem,

(13) $$d_{j}\left(\lambda_{j}^{k}\right)=\{\begin{array}{cc} \underset{r_{j},y_{j}^{k}}{\text{max}} & U_{j}\left(r_{j}\right)-\lambda_{j}^{k}y_{j}^{k}\\ s.t. & m_{j}\leq r_{j}\leq M_{j}\\ & r_{j}\leq y_{j}^{k} \end{array}$$

Note that Problem (13) coincides with Problem (8) if we take Equation (10) into account and thus we keep the nomenclature, that is, we refer to Problem (13) as the dual subproblems of the CDM. As a result of computing the dual subproblems, the optimal values of the inner optimization variables $y_{j}^{k}$ given $\lambda_{j}^{k}$, *i.e.*, $y_{j}^{*,k}(\lambda_{j}^{k})$ are found as well. However, these values are not necessarily primal feasible, *i.e.*, they may not satisfy ***Ay*** ≼ ***c***, and there is no guarantee that they accomplish the necessary slackness conditions in Equation (11). In the second step of the method, called the primal projection, we modify the resulting allocation ***y****^k^* in order to satisfy both conditions.

#### 2.4.2. Primal Projection

**Figure 3.**
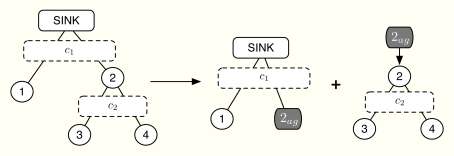
Distributed primal projection. Initially, nodes 2, 3 and 4 are grouped together into the supernode 2’. The first allocation distributes *c*_1_ between 1 and 2’. Then, the second allocation distributes the rate granted to 2’ among nodes 2, 3 and 4 taking into account the maximum rate *c*_2_.

In primal projection, the following optimization problem is solved,

(14) $$\begin{array}{lll} \underset{{\hat{y}}^{k}}{\text{min}} & {\left\|\left(y^{k}-{\hat{y}}^{k}\right)\right\|}^{2} & \\ s.t. & {\left[A\right]}_{j}{\hat{y}}^{k}\leq c_{j} & \forall_{j}|\mu_{j}^{k}=0\\ & {\left[A\right]}_{j}{\hat{y}}^{k}=c_{j} & \forall_{j}|\mu_{j}^{k}>0 \end{array}$$

and we get the modified variables ***ŷ****^k^* as the optimal solution of Problem (14). As it is discussed in Section 2.8, the algorithm guarantees that Problem (14) has at least one feasible point so that Problem (14) is well-defined. However, we need a distributed solution of the primal projection in order to avoid sending all the information to the sink node and computing Problem (14) there. Fortunately, we can benefit from the tree structure of the network to find the optimal solution of Problem (14) distributedly. Note that in cluster-tree topologies, the subset of nodes associated with the i-th constraint is always a subset of the nodes related to the constraint immediately above *c_i_*. See this in the small network example of Figure 3, where

(15) $$\left[\begin{array}{c} c_{1}\\ c_{2} \end{array}\right]≽\left[\begin{array}{cccc} 1 & 1 & 1 & 1\\ 0 & 0 & 1 & 1 \end{array}\right]\left[\begin{array}{c} {\hat{y}}_{1}\\ {\hat{y}}_{2}\\ {\hat{y}}_{3}\\ {\hat{y}}_{4} \end{array}\right]$$

For the sake of brevity, we describe the distributed computation of Problem (14) using the example in Figure 3, which can be easily extended to the general case. Let us assume that [***A***]_1_ ***ŷ*** = *c*_1_ (this is usually the case) and [***A***]_2_ ***ŷ*** ≤ *c*_2_ [31]. For the sake of simplicity, we omit the index *k* in this example. As depicted in Figure 3, the idea is to compute the projection at the parent nodes, that is, first the sink allocates *ŷ*_1_ and *ŷ*_2,*ag*_ and thereafter, sensor 2 allocates *ŷ*_2,*ag*_ to sensors 3–4 and itself, thus obtaining *ŷ*_2_, *ŷ*_3_ and *ŷ*_4_. On the other hand, the application of the KKT optimality conditions to our simplified problem reveals us that the optimal solution must satisfy

(16) $$\begin{array}{ll} {\hat{y}}_{j}=y_{j}-\mu_{1}, & j=1,2\\ {\hat{y}}_{k}=y_{k}-\left(\mu_{1}+\mu_{2}\right), & k=3,4 \end{array}$$

where *μ*_1_ ∈ ℝ forces ∑_*i*_ *ŷ_i_* = *c*_1_ and *μ*_2_ ∈ ℝ^+^ ∪ {0} prevents *ŷ*_3_ + *ŷ*_4_ from exceeding *c*_2_. In other words, *μ*_2_ is bigger than 0 only when the constraint *ŷ*_3_ + *ŷ*_4_ ≤ *c*_2_ is violated given *μ*_1_. Therefore, there are only two possible projections, that is, either i) *ŷ*_3_ + *ŷ*_4_ *< c*_2_ or ii) *ŷ*_3_ + *ŷ*_4_ = *c*_2_. Let us consider a virtual sensor 2*_ag_* that groups sensors 2–4 with $y_{2,\mathrm{ag}}=\sum_{i=2}^{4}y_{i}$ as in Figure 3. In case (i), the projection is straightforwardly computed [18] (Section 8.1.1) as

(17) $${\hat{y}}_{\mathrm{ag}}=y_{\mathrm{ag}}-\frac{\left(1^{T}y_{\mathrm{ag}}-c_{1}\right)n_{\mathrm{ag}}}{1^{T}n_{\mathrm{ag}}}$$

where ***ŷ****_ag_* = [*ŷ*_1,*ag*_, *ŷ*_2,*ag*_]*^T^* is the aggregated allocation to the virtual sensors, ***y****_ag_* = [*y*_1,*ag*_, *y*_2,*ag*_]*^T^* is their aggregated demand and ***n****_ag_* = [*n*_1,*ag*_, *n*_2,*ag*_]*^T^* is the number of sensors inside each virtual sensor. In our example, ***n****_ag_* = [1, 3]*^T^*. However, if (ii) is the case, we allocate *c*_1_ − *c*_2_ to sensors 1–2 similarly as in Equation (17), as well as *c*_2_ to sensors 3–4. Finally, we need to distinguish between situations (i) and (ii), which can be done using the following threshold for virtual node 2*_ag_*,

(18) $${\hat{y}}_{2,\mathrm{ag}}^{\mathrm{th}}=y_{2,\mathrm{ag}}-\frac{n_{2,\mathrm{ag}}}{n_{2,\mathrm{ag}}-1}\left(y_{2,\mathrm{ag}}-y_{2}\right)$$

This value is the minimum necessary allocation to virtual node 2*_ag_* so that the inner rate constraint, *i.e.*, *ŷ*_3_ + *ŷ*_4_ ≤ *c*_2_, is satisfied with equality.

In summary, the primal projection is distributedly found as follows. First, we compute the initial allocation with Equation (17). If ${\hat{y}}_{2,\mathrm{ag}}\leq {\hat{y}}_{2,\mathrm{ag}}^{\mathrm{th}}$, then we allocate *ŷ*_2,*ag*_ to sensors 2–4 and we are done. If not, we allocate *c*_1_ − *c*_2_ to sensors 1–2 and *c*_2_ to sensors 3–4. Finally, this approach can be scaled to any tree-deployed sensor network. In general, a parent node has to signal at most *m_l_* 4-tuples, where *m_l_* is the number of network levels below it. Each 4-tuple contains an aggregated rate demand, the number of nodes involved in that demand, a threshold value and the rate absorbed once the allocation is above it. Note that this threshold value indicates the maximum possible allocation for a given hypothesis, that is, which channels are congested and which not. Therefore, the absorbed rate must correspond to this specific hypothesis. Once this information is available at the sink node, it can make the first-level allocation and successively, all parent nodes can compute their own allocations.

#### 2.4.3. Primal Subproblems

Once the sensors receive the corrected allocation ${\hat{y}}_{j}^{k}$, they solve the following optimization problem,

(19) $$p_{j}\left({\hat{y}}_{j}^{k}\right)=\{\begin{array}{cc} \underset{r_{j}}{\text{max}} & U_{j}\left(r_{j}\right)\\ s.t. & m_{j}\leq r_{j}\leq M_{j}\\ & r_{j}\leq {\hat{y}}_{j}^{k} \end{array}$$

As above, note that Problem (19) coincides with the subproblems of a plain primal decomposition [22], thus we keep the nomenclature. As a result of computing Problem (19), the inner Lagrange multiplier ${\hat{\lambda }}_{j}^{k}$ associated with the constraint $r_{j}\leq {\hat{y}}_{j}^{k}$ is obtained. In the dual projection, we use the values of ${\hat{\lambda }}_{j}^{k}$ from all the subproblems to update the values of ***μ***, that is, to obtain ***μ***^*k*+1^.

#### 2.4.4. Dual Projection

The dual projection is the last step of the method and its mission is to update ***μ*** having the information in ***λ̂**^k^*. We already know that one of the optimality conditions of our problem is Equation (10). However, this linear system of equations is in general overdetermined and we can not use it to guess ***μ***^*k*+1^ from ***λ̂**^k^*. Fortunately, it is possible to choose some of the entries in ***λ̂**^k^* to obtain an alternative system of equations, say $\lambda_{D}^{k}=B_{D}^{k}\mu_{D}^{k+1}$, that determines the non-zero values in ***μ***^*k*+1^. Note that due to Equation (11), $\mu_{j}^{k+1}$ is necessarily 0 if the corrected allocation after the primal projection verifies [***A***]*_j_****ŷ****^k^* − *c_j_ <* 0. In the following, we describe how $\lambda_{D}^{k}$ and $B_{D}^{k}$ are constructed.

**Figure 4.**
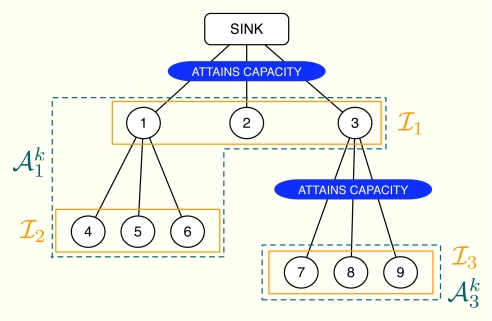
The subsets 𝒤*_m_* and ${𝒜}_{n}^{k}$.

First of all, let us define the subsets of nodes 𝒤*_m_* and ${𝒜}_{n}^{k}$. The subset 𝒤*_m_* does not depend on the iteration number and includes all the nodes using the m-th wireless channel except for the parent node. On the contrary, ${𝒜}_{n}^{k}$ depends on the subset of active constraints [32] in the network, which evolves on time. Specifically, if the n-th channel is congested, the subset ${𝒜}_{n}^{k}$ is the union of 𝒤*_n_* with all the subsets 𝒤*_m_* that accomplish: (i) the path from any of the nodes in 𝒤*_m_* to the sink goes through one of the nodes in 𝒤*_n_* and (ii) none of these paths makes use a congested channel, that is, the channels in the path do not attain their rate constraints with equality. If the n-th channel is not congested, we do not define ${𝒜}_{n}^{k}$. A simple example can be found in Figure 4, where 𝒤_1_ = {1, 2, 3}, 𝒤_2_ = {4, 5, 6} and 𝒤_3_ = {7, 8, 9}. In this example, we transmit at the maximum possible rate only in channels 1 and 3 whereas channel 2 is not congested. Therefore, we define ${𝒜}_{1}^{k}={𝒤}_{1}\cup {𝒤}_{2}$ and ${𝒜}_{3}^{k}={𝒤}_{3}$ but not **𝒜**_2_.

**Figure 5.**
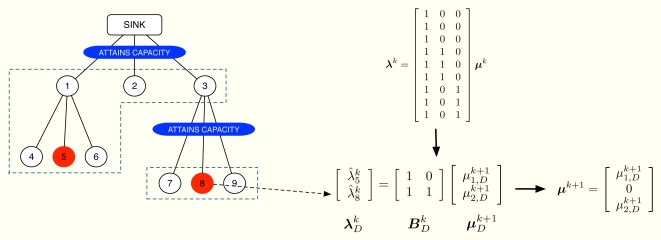
Dual projection example. The reduced system of equations $\lambda_{D}^{k}=B_{D}^{k}\mu_{D}^{k+1}$ is constructed and used in the dual projection to update the non-zero values in ***μ***^*k*+1^.

Once the subsets { ${𝒜}_{n}^{k}$} are identified, we select one sensor from each group using the following selection rules:

(SR1) Choose the node with the value of ${\hat{\lambda }}_{j}^{k}$ that is closest to $\lambda_{j}^{k}$ (note that $\lambda_{j}^{k}=\lambda_{i}^{k}$ if *i*, $j\in {𝒜}_{n}^{k}$) and that verifies (SR2).
(SR1) The corresponding value in ${\hat{y}}_{j}^{k}$ attains ${\hat{y}}_{j}^{k}\in (m_{j},M_{j})$ (as shown in Proposition 3, there is at least one value of ${\hat{y}}_{j}^{k}$ inside the interval).

(SR1) forces the smallest update from $\lambda_{j}^{k}$ to $\lambda_{j}^{k+1}$ whereas (SR2) is necessary to have the required linear dependance between ***λ*** and ***μ***; otherwise there exist more dual variables that come into the equation as shown in Section 2.8. Finally, the linear system of equations $\lambda_{D}^{k}=B_{D}^{k}\mu_{D}^{k+1}$ is constructed by gathering all the selected values of **λ̂***^k^* in $\lambda_{D}^{k}$ and the corresponding rows of ***A****^T^* in $B_{D}^{k}$. Note that since $\mu_{D}^{k+1}$ does not include the values of ***μ***^*k*+1^ that are known to be zero beforehand, it is necessary to discard the corresponding rows of ***A****^T^* when building $B_{D}^{k}$. An example can be found in Figure 5, where we assume that the selected value from ${𝒜}_{1}^{k}$ is ${\hat{\lambda }}_{5}^{k}$ and that the selected value from ${𝒜}_{3}^{k}$ is ${\hat{\lambda }}_{8}^{k}$. Therefore, we take into account only rows 5 and 8 of ***A****^T^* in order to obtain $B_{D}^{k}$. Furthermore, since channel 2 is not congested (that’s why we have not defined ${𝒜}_{2}^{k}$), we already know that $\mu_{2}^{k+1}=0$. Consequently, we also discard the second column of ***A****^T^*. The resulting linear system of equations is determined and allows us to obtain $\mu_{1}^{k+1}=\mu_{1,D}^{k+1}$ and $\mu_{3}^{k+1}=\mu_{2,D}^{k+1}$. However, we want ***μ***^*k*+1^ to verify Equation (12) and thus, we propose next a distributed approach to compute ***μ***^*k*+1^ and ***λ***^*k*+1^ in a cluster-tree WSN. Our mechanism solves $\lambda_{D}^{k}=B_{D}^{k}\mu_{D}^{k+1}$ if $\mu_{D}^{k+1}≽0$ and finds an alternative positive solution otherwise, which guarantees the convergence of the method (see Section 2.8). Note that, due to the specific structure of ***A****^T^* and also of $B_{D}^{k}$, we can apply some sort of Gauss elimination as discussed next.

The proposed solution has two phases. The first phase begins at the lowest levels of the cluster-tree and each node sends its current value of ${\hat{\lambda }}_{j}^{k}$ to its parent. The parent, in turn, first checks if the channel with its child nodes is congested. If true, it sends its own value of ${\hat{\lambda }}_{j}^{k}$. If false, it takes the values from the child nodes and also its own value and selects one candidate according to (SR1)-(SR2). This is the value that is sent to the parent in this second case. This process is successively applied until the sink node is reached. Thereafter, the second phase begins. The sink node checks if it attains its own rate constraint. If true, it selects one value taking into account (SR1)-(SR2), fixes *μ*_1_ to that value and sends it to its child nodes. If false, it fixes *μ*_1_ = 0 and sends it. When the child nodes receive that value, say *v_i_*, select one ${\hat{\lambda }}_{j}^{k}$ among its gathered values using again (SR1)-(SR2) and compute *μ_i_* (the dual multiplier associated with its rate constraint) as $\mu_{i}={\hat{\lambda }}_{j}^{k}-v_{i}$. If the resulting *μ_i_* ≥ 0, the parent node sends ${\hat{\lambda }}_{j}^{k}$ to its child nodes; if *μ_i_* < 0 or if the channel between the node and its children is not congested, the parent fixes *μ_i_* = 0 and sends the received *v_i_* to its child nodes. This process is repeated until all the values in ***μ***^*k*+1^ are updated. Note that, as a result of the proposed distributed dual projection, each parent node only knows one value in ***μ***^*k*+1^ but each node receives its own $\lambda_{j}^{k+1}$ so that the next iteration of the method can begin.

Finally, we have to decide when to stop the iterations of the CDM, that is, when the current solution is close enough to the optimum. One possibility is to monitor the distance between ***y****^k^* and ***ŷ****^k^* and to stop when the relative distance between both quantities drops below a predefined value *ε*, that is, when

(20) $$\frac{{\left\|y^{k}-{\hat{y}}^{k}\right\|}^{2}}{{\left\|{\hat{y}}^{k}\right\|}^{2}}<\varepsilon$$

However, this approach requires some signalling because we do not have all the necessary data available at a single node. An alternative approach with a very reduced signalling, which is preferred, is to compute the same relative distance at the sink node taking into account aggregated rate values of the child nodes. In this case, we use the previous definitions of $y_{j,\mathrm{ag}}^{k}$ as the aggregated allocation demand (before the primal projection) at the j-th node and the k-th iteration and ${\hat{y}}_{j,\mathrm{ag}}^{k}$ as the aggregated allocation, *i.e.*, after the primal projection. For example, in the small network in Figure 3, we have $y_{1,\mathrm{ag}}^{k}=y_{1}^{k}$ and $y_{2,\mathrm{ag}}^{k}=\sum_{j=2}^{4}y_{j}^{k}$. Then, we collect at the sink node the values of $y_{j,\mathrm{ag}}^{k}$ and ${\hat{y}}_{j,\mathrm{ag}}^{k}$ for all its child nodes in the vectors $y_{\mathrm{ag}}^{k}$ and ${\hat{y}}_{\mathrm{ag}}^{k}$, respectively. Using these vectors, we compute the relative distance between them as in In equation (20). Note that using this approach, we do not introduce additional signalling as far as these quantities are necessary in the primal projection of the method. Furthermore, the decision to stop or continue with the iterations of the CDM can be piggybacked in the messages that send the values of ${\hat{y}}_{j_{\mathrm{ag}}}^{k}$ from the parent nodes to the child nodes and thus, the impact in the signalling requirements of the method is very small.

### 2.5. Algorithmic Form

The CDM can be summarized in algorithmic form as follows:

**Algorithm.** Coupled-Decompositions Method.

Start with *k* = 0, ***μ***^0^ = **0** and repeat:
*Dual Subproblems*
1⃞ Using $\lambda_{j}^{k}={\left[A^{T}\right]}_{j}\mu^{k}$, compute $$d_{j}\left(\lambda_{j}^{k}\right)=\{\begin{array}{cc} \underset{r_{j},y_{j}^{k}}{\text{max}} & U_{j}(r_{j})-\lambda_{j}^{k}y_{j}^{k}\\ s.t. & m_{j}\leq r_{j}\leq M_{j}\\ & r_{j}\leq y_{j}^{k} \end{array}$$ and get $y_{j}^{k}$ as the inner maximizer.
*Primal Projection*
2⃞ Solve $$\begin{array}{ccc} \underset{{\hat{y}}^{k}}{\text{min}} & {\left\|\left(y^{k}-{\hat{y}}^{k}\right)\right\|}^{2} & \\ s.t. & {\left[A\right]}_{j}{\hat{y}}^{k}\leq c_{j} & \forall_{j}|\mu_{j}^{k}=0\\ & {\left[A\right]}_{j}{\hat{y}}^{k}=c_{j} & \forall_{j}|\mu_{j}^{k}>0 \end{array}$$ distributedly as sketched in Section 2.4 in order to get ${\hat{y}}_{j}^{k}$.
*Primal Subproblems*
3⃞ Solve $$p_{j}\left({\hat{y}}_{j}^{k}\right)=\{\begin{array}{cc} \underset{r_{j}}{\text{max}} & U_{j}(r_{j})\\ s.t. & m_{j}\leq r_{j}\leq M_{j}\\ & r_{j}\leq {\hat{y}}_{j}^{k} \end{array}$$ and get ${\hat{\lambda }}_{j}^{k}$ as the dual multiplier associated with the constraint $r_{j}\leq {\hat{y}}_{j}^{k}$.
*Dual Projection*
4⃞ *Phase I*. The lowest level nodes send ${\hat{\lambda }}_{j}^{k}$ to their parents. Then repeat: Each parent node checks the congestion status of the channel between it and its children. If it is congested, the parent sends its own value of ${\hat{\lambda }}_{j}^{k}$ to its own parent. Otherwise, it considers also the values received from its child nodes, selects one candidate using (SR1)-(SR2) and sends it. Until the sink node has received one value of ${\hat{\lambda }}_{j}^{k}$ from each of its child nodes. *Phase II*. Start at the sink node with *v*_1_ = 0 and repeat: If the channel between the node and its children is congested, use (SR1)-(SR2) to select one candidate among the values received in Phase I, say ${\hat{\lambda }}_{j}^{k}$. Otherwise, ${\hat{\lambda }}_{j}^{k}=0$. Compute $\mu_{i}^{k+1}={\hat{y}}_{j}^{k}-v_{i}$ (*v_i_* is the message received from the parent node in Phase II, *i* is the channel index). If the resulting value $\mu_{i}^{k+1}$ satisfies $\mu_{i}^{k+1}\geq 0$, send ${\hat{\lambda }}_{j}^{k}$ to the child nodes. Otherwise, fix $\mu_{i}^{k+1}=0$ and send *v_i_*. Set the next update of *λ_j_* at the child nodes, *i.e.*, $\lambda_{j}^{k+1}$, to the received value, either ${\hat{\lambda }}_{j}^{k}$ or *v_i_*. Until all the values in ***μ****^k^* are determined and all the values in ***λ****^k^* are updated to ***λ***^*k*+1^.
Until convergence.

**Figure 6.**
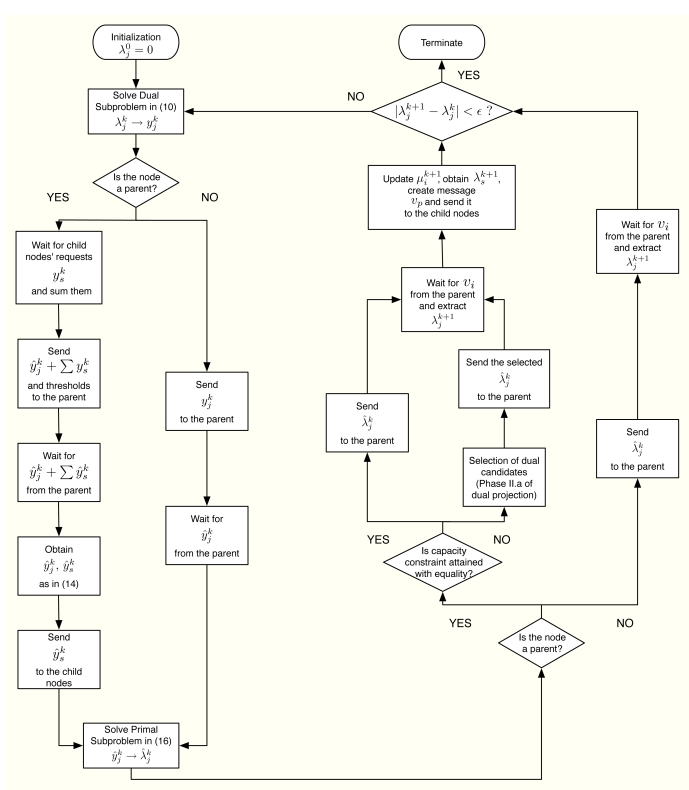
Flowchart of the tasks performed by the sensor.

Furthermore, Figure 6 summarizes in a flowchart all the tasks that a sensor has to execute in order to obtain its optimal rate allocation.

### 2.6. Signalling in the Coupled-Decompositions Method

**Figure 7.**
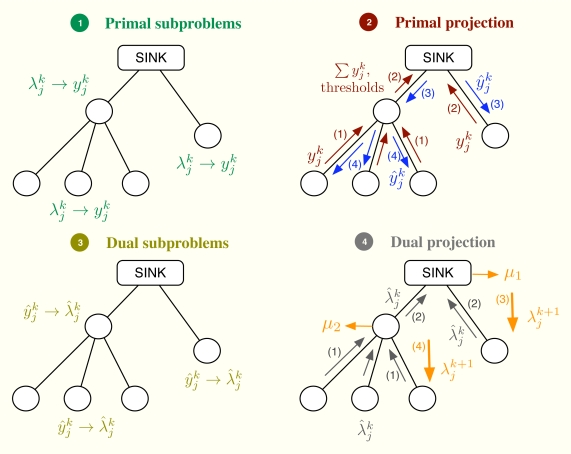
A complete iteration of the CDM from the point of view of signalling.

In distributed implementations such as the one proposed in this paper, signalling plays a crucial role. In the following, we review a complete iteration of the proposed method from the point of view of signalling and we represent it graphically in Figure 7. Let us assume that at the k-th iteration, all the nodes know the values of $\lambda_{j}^{k}$ and use them to compute the dual subproblems. As a result, they locally obtain the values of $y_{j}^{k}$ involving no signalling. Thereafter, in primal projection, the parent nodes must send their aggregated request $y_{j,\mathrm{ag}}^{k}$, *i.e.*, the sum of the values $y_{j}^{k}$ that belong to their subtree, possibly together with a list that includes allocation threshold values and absorbed rates, as discussed in Equations (17) and (18). Note that the size of the list scales linearly with the number of levels in the subtree. Once the request reaches the sink node, it allocates resources to the first level of the tree. In turn, the nodes at the first level allocate their resources to the nodes at the second level and so forth. When all the nodes have received the corresponding value ${\hat{y}}_{j}^{k}$, they locally compute the primal subproblems and obtain the values ${\hat{\lambda }}_{j}^{k}$. Finally, in order to compute the dual projection, the nodes send the values ${\hat{\lambda }}_{j}^{k}$ to their parent nodes. Thereafter, the sink node computes *μ*_1_ and broadcasts the value $\lambda_{j}^{k+1}$ to their child nodes (note that all the child nodes receive the same value). In turn, the parent nodes compute their own value *μ_j_* and broadcast $\lambda_{j}^{k+1}$ to their child nodes. This process is successively applied until all the nodes receive the updated value of the dual variable, which completes one iteration of the proposed CDM.

### 2.7. Differences with Classical Decompositions

From the point of view of the algorithm, two of the building blocks in the proposed method are the same as in the classical decompositions, that is, the definition of the primal and the dual subproblems has not been modified. On the contrary, the primal and the dual projections differ from the primal and dual master problems as far as subgradients are no longer used. Notwithstanding, the primal projection resembles the projection that appears in the primal master problem. The difference is that we force the constraints in ***Ay*** ≼ ***c*** to be attained with equality when the corresponding dual variable in ***μ*** is not zero. Finally, the dual projection is completely different from the master dual problem and is indeed the key block in our proposal.

From the point of view of signalling, one iteration in traditional primal decomposition algorithms is equivalent to the first two steps in the proposed method. In other words, from $y_{j}^{k}$ the nodes compute the primal subproblems and calculate $y_{j}^{k+1}=y_{j}^{k}+\alpha_{p}^{k}\lambda_{j}^{k}$, which requires no signalling. Thereafter, the projection [·]^†^ in traditional primal decomposition algorithms requires the same amount of signalling as in the worst-case of the primal projection at the CDM, that is, when [***A***]*_j_****y*** ≼ *c_j_*, ∀*j*. Similarly, one iteration in the traditional dual decomposition is equivalent to the last two steps of the CDM. That is, from ***μ***^*k*^, the nodes implementing the traditional dual decomposition approach obtain $y_{j}^{k}$, which requires no signalling, and afterwards send these values to the parent nodes. Using that information the parent nodes can extract the values of ***μ***^*k*+1^ and send the required linear combination of the values in ***μ***^*k*+1^ back to the child nodes. In this case, the total amount of signalling is exactly the same as in the dual projection of the CDM, where the nodes send the current allocation, the parent nodes compute the required subgradient and send the updates of the values in ***λ*** afterwards.

In summary, the key step that allows us to combine traditional primal and dual decompositions in a single algorithm, the CDM, is the dual projection. Conceptually, the big difference is to interpret the primal variables ***y****^k^*, ***ŷ****^k^* and the dual variables ***λ**^k^*, **λ̂***^k^* as candidates to the optimal solution that must accomplish the well-known KKT optimality conditions. In terms of signalling, one iteration of the CDM is equivalent to two iterations of the traditional decomposition algorithms.

### 2.8. Convergence to the Optimal Rate Allocation

Let us start the iterations of the proposed CDM with ***μ***^0^ = **0**. In the following we show that ***μ****^k^* → μ* as *k* increases. Initially, we assume that at the k-th iteration $\mu_{i}^{k}>0$ only if $\mu_{i}^{*}>0$, that is, the active constraints (*i.e.*, attained with equality) at the k-th iteration are exactly the same as in the optimal solution and we prove that the algorithm converges to μ*. Afterwards, we describe the mechanism used by the CDM in order to activate or deactivate constraints until the subset of active constraints in the optimal solution is found out.

#### 

##### Convergence to the Optimal Solution Given the Subset of Active Constraints at the Optimum

Let us first recall in Proposition 1, a known result that establishes the relationship between primal and dual variables in the primal and dual subproblems [11,12].

**Proposition 1** *Let us consider the j-th primal subproblem* $p_{j}({\hat{y}}_{j}^{k})$ *in Problem (19) and the j-th dual subproblem* $d_{j}(\lambda_{j}^{k})$ *in Problem (13) of the CDM. Then, the inner optimization variable* ${\hat{\lambda }}_{j}^{k}$ *is a decreasing function of* ${\hat{y}}_{j}^{k}$ *in the primal subproblems and the inner optimization variable* $y_{j}^{k}$ *is a decreasing function of* $\lambda_{j}^{k}$ *in the dual subproblems.*

**Proof** See [12] (Lemma 2).

Note, in particular, that if ${\hat{\lambda }}_{j}^{k}\leq \lambda_{j}^{*}=A^{T}\mu^{*}$ as a result of the primal subproblems, then it is true that ${\hat{y}}_{j}^{k}\geq y_{j}^{*}$ and vice versa. Similarly, if $y_{j}^{k}\leq y_{j}^{*}$ as a result of the dual subproblems, then $\lambda_{j}^{k}\geq \lambda_{j}^{*}=A^{T}\mu^{*}$ and vice versa. Note also that given ***μ****^k^*, all the values $\lambda_{j}^{k}={\left[A^{T}\right]}_{j}\mu^{k}$ with *j* ∈ 𝒤*_i_* attain either $\lambda_{j}^{k}\geq \lambda_{j}^{*}$ or $\lambda_{j}^{k}\leq \lambda_{j}^{*}$ as far as [***A****^T^*]*_j_* is exactly the same for all *j* ∈ 𝒤*_i_*. That is, if we start an iteration of the CDM with ***λ**^k^* in the dual subproblems, it holds that $y_{j}^{k}\geq y_{j}^{*}$ for all *j* ∈ 𝒤*_k_* if $\lambda_{j}^{k}\leq \lambda_{j}^{*}$ and vice versa due to Proposition 1.

Once we obtain the values ***y****^k^* from the dual subproblems, the primal projection in Problem (14) is computed. Since we assume that Slater’s condition holds, Problem (14) defines a convex program where strong duality is fulfilled. Therefore, KKT optimality conditions can be applied and the solution to the primal projection can be expressed as

(21) $${\hat{y}}^{k}=y^{k}-A^{T}\gamma$$

where *γ_i_* ≥ 0 if [***A***]*_i_****ŷ*** ≤ *c_i_* and *γ_j_* ∈ ℝ if [***A***]*_j_****ŷ***= *c_j_*. In the first case, *γ_i_* = 0 if the corresponding rate constraint is not exceeded and *γ_i_ >* 0 if the opposite situation occurs in order to satisfy it with equality. In the second case, *γ_j_* can take any value in order to satisfy the corresponding constraint. As a result of the primal projection, it is clear that all the nodes in every subset 𝒤*_i_* either increase or decrease the initial allocation $y_{j}^{k}$ in order to obtain ${\hat{y}}_{j}^{k}$. In other words, if *m*, *n* ∈ 𝒤*_i_*, then it is impossible, for example, that ${\hat{y}}_{m}^{k}=y_{m}^{k}-a_{1}$ and ${\hat{y}}_{n}^{k}=y_{n}^{k}+a_{2}$ with *a*_1_, *a*_2_ ≥ 0. Moreover, if ***μ****^k^* is close enough to the optimal solution and assuming that $\mu_{j}^{k}>0$ only if $\mu_{j}^{*}>0$, the constraints that are attained with equality after the primal projection correspond only to the values $\mu_{j}^{k}>0$.

In that situation and given the tree structure of the network, it is verified that

(22) $$\sum_{j\in {𝒜}_{m}^{k}}{\hat{y}}_{j}^{k}=\sum_{j\in {𝒜}_{m}^{k}}y_{j}^{*}$$

For example, in Figure 4 we immediately notice that $\sum_{j\in {𝒜}_{3}^{k}}{\hat{y}}_{j}^{k}=c_{3}$ and $\sum_{j\in {𝒜}_{1}^{k}}{\hat{y}}_{j}^{k}=c_{1}-c_{3}$. Therefore, we can conclude that i) some values of ${\hat{y}}_{j}^{k}$ with $j\in {𝒜}_{m}^{k}$ attain ${\hat{y}}_{j}^{k}\geq y_{j}^{*}$ while the rest accomplish ${\hat{y}}_{j}^{k}\leq y_{j}^{*}$ and ii) all the values ${\hat{y}}_{j}^{k}$ with $j\in {𝒜}_{m}^{k}$ either increase or decrese after the primal projection. This result is easily extrapolated to the dual values ${\hat{\lambda }}_{j}^{k}$ obtained from the primal subproblems when ${\hat{y}}_{j}^{k}$ are the inputs if we take into account Proposition 1. We write it down in the following proposition.

**Proposition 2** *The dual values* ${\hat{\lambda }}_{j}^{k}$ *that result from the computation of the primal subproblems of the CDM at the k-th iteration accomplish: (i) some values of* ${\hat{\lambda }}_{j}^{k}$ *with* $j\in {𝒜}_{m}^{k}$ *attain* ${\hat{\lambda }}_{j}^{k}\geq \lambda_{j}^{*}$ *while the rest accomplish* ${\hat{\lambda }}_{j}^{k}\leq \lambda_{j}^{*}$ *and (ii) all the values* ${\hat{\lambda }}_{j}^{k}$ *with* $j\in {𝒜}_{m}^{k}$ *either increase or decrease with respect to* $\lambda_{j}^{k}$ *(note that* $\lambda_{l}^{k}=\lambda_{n}^{k}$ *if l,* $n\in {𝒜}_{m}^{k}$*).*

In the dual projection, we choose one value ${\hat{\lambda }}_{j}^{k}$ from each subset ${𝒜}_{m}^{k}$. In particular, the selected value has to accomplish (SR1)-(SR2), that is: (i) the corresponding ${\hat{y}}_{j}^{k}$ verifies ${\hat{y}}_{j}^{k}\in (m_{j},M_{j})$ and (ii) it is the closest value in the subset to $\lambda_{j}^{k}$ for any $j\in {𝒜}_{m}^{k}$. The following proposition guarantees the existence of the required value.

**Proposition 3** *Let* ***y****^k^ be the result of the dual subproblems of the CDM at the k-th iteration. Then, the primal projection provides at least one node with* ${\hat{y}}_{j}^{k}\in (m_{j},M_{j})$ *at each subset* ${𝒜}_{m}^{k}$.

**Proof** See the Appendix.

Given that *U_j_*(*r_j_*) is concave and strictly increasing, it is straightforward to see that *y_j_* must equal *r_j_* in Problem (3). Furthermore, some of the KKT optimality conditions of the problem reveal us that

(23) $$\frac{\partial U_{j}}{\partial r_{j}}-\lambda_{j}+\alpha_{j}-\beta_{j}=0$$

(24) $$\lambda_{j}-{\left[A^{T}\right]}_{j}\mu =0$$

(25) $$\alpha_{j}\left(m_{j}-r_{j}\right)=0$$

(26) $$\beta_{j}\left(M_{j}-r_{j}\right)=0$$

where *α_j_* and *β_j_* are the dual variables associated with the constraints *r_j_* ≥ *m_j_* and *r_j_* ≤ *M_j_*, respectively. Therefore, if we fix *y_j_* equal to ${\hat{y}}_{j}^{k}$, with ${\hat{y}}_{j}^{k}\in (m_{j},M_{j})$, then *α_j_* and *β_j_* must be 0 due to the slackness constraints, *i.e*, Equations (25–26). Applying this observation and $r_{j}={\hat{y}}_{j}^{k}$ to Equation (23), we realize that *λ_j_* is uniquely determined. Besides, taking also Equation (24) into account, we can conclude that a good election of ${\hat{y}}_{j}^{k}$ provides us useful information about the linear combination [***A****^T^*]*_j_****μ***. In the CDM, this argumentation justifies our interest in the values of ${\hat{y}}_{j}^{k}$ that attain ${\hat{y}}_{j}^{k}\in (m_{j},M_{j})$. Otherwise the values of *α_j_* and *β_j_* are unknown and we can not use ${\hat{\lambda }}_{j}^{k}$ to determine ***μ***^*k*+1^.

Finally, in the dual projection we construct the determined linear system $\lambda_{D}^{k}=B_{D}^{k}\mu_{D}^{k+1}$ with the selected values ${\hat{\lambda }}_{j}^{k}$, thus computing the values in ***μ***^*k*+1^ corresponding to the active constraints (the remaining ones are fixed to zero). Combining the results in Proposition 2 with (SR1)-(SR2), we realize that each dual candidate ${\hat{\lambda }}_{j}^{k}$ approaches $\lambda_{j}^{*}$ (w.r.t. $\lambda_{j}^{k}$) without overtaking it and that the candidate is valid to determine some of the variables in ***μ***^*k*+1^. Since in the dual projection we obtain a determined linear system using these dual candidates, the new update ***λ**^k+1^* = [***A***]*^T^* ***μ***^*k*+1^ necessarily approaches ***λ**^k^* = [***A***]*^T^* ***μ****^k^*. Hence, we can state that ***μ****^k^* → μ*. We summarize this result in the following lemma.

**Lemma 1** *The iterations of the CDM satisfy* $\mu^{k}\overset{k\to \infty }{\to }\mu *$ *given the subset of active constrains at the optimum.*

##### Activation/Deactivation of Constraints

In the following, we use the partial result obtained in Lemma 1 to show that the proposed version of the CDM converges to the optimal solution. Let us state this in the following theorem.

**Theorem 1** *The proposed CDM is able to determine the subset of active constraints of Problem (3) at the optimal solution. Hence, the method converges to the optimal solution.*

**Proof** We have proved in Lemma 1 that, given the subset of active constraints at the optimum, the CDM converges to μ*. Therefore, we need to prove that this subset is eventually found out. Let us start with the activation of a non-active constraint and let us assume that the l-th constraint is active at the optimal solution but not at the k-th iteration. This implies that, if we remove the constraint [***A***]*_l_****y*** ≤ *c_l_* from the original problem formulation in Problem (3), then the optimal solution to the modified problem ***y***^‡^ would satisfy [***A***]*_l_****y***^‡^ ≥ *c_l_*. In other words, the successive updates of the CDM tend to ***y***^‡^ when the l-th constraint is not included in the active subset of constraints. However, there will be an iteration *q* where [***A***]*_l_****y****^q^* ≥ *c_l_* so that the primal projection will detect that constraint violation, correct it and the l-th constraint will be activated.

The opposite situation arises when the l-th constraint is active at the k-th iteration but not at the optimal solution. In that case, if the l-th constraint is not deactivated, *i.e.*, the primal projection keeps forcing ***Ay*** = *c_l_*, the CDM would converge to a solution ***μ*^★^** with $\mu_{l}^{★}<0$ (assuming that the dual projection allows negative values). Note that the allocation to the variables involved in the l-th constraint increases with respect to the optimum whereas the allocation to the remaining variables is reduced or remains unchanged in order to satisfy the rate constraints. Looking at the situation from the dual point of view, that is, taking into account Proposition 1, we notice that the values $\lambda_{j}^{★}$ involved in the l-th constraint must converge to a value that is below $\lambda_{j}^{*}$. Also, the remaining values remain unchanged or above the corresponding $\lambda_{j}^{*}$. If we compare those values with λ* = ***A****^T^* μ* and we know that $\mu_{l}^{*}=0$, then we realize that $\mu_{l}^{★}<0$. At this point, let us compute one more iteration of the CDM, thus obtaining ***λ***^**★**+1^. In this case, since the values $\lambda_{j}^{★+1}$ involved in the l-th constraint increase with respect to **λ^★^** = ***A****^T^* ***μ*^★^** because $\mu_{l}^{★+1}$ is now 0, the corresponding values $y_{j}^{★+1}$ decrease. Therefore the l-th rate constraint will not be exceeded, preventing its reactivation at the next primal projection.

## 3. Application to Cluster-Tree Wireless Sensor Networks

**Figure 8.**
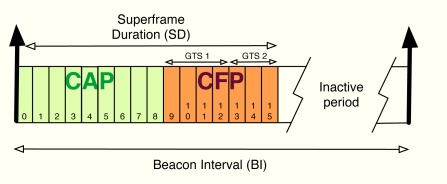
Beacon frame configuration adopted by the IEEE 802.15.4 standard [1].

**Figure 9.**
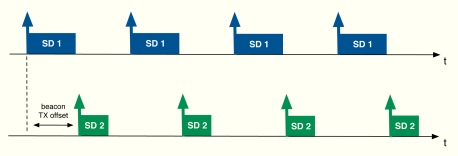
Beacon frame collision avoidance based on the time division approach adopted by the Zigbee specification [9].

The algorithm developed in the previous section can be applied to cluster-tree WSNs with the aim of providing global fair resource allocations with a reduced impact in terms of energy consumption due to signalling. In this work, in particular, we focus on the beacon-enabled mode of IEEE 802.15.4 [1]. This mode divides the transmission through time slots and, besides, two periods are defined depending on the kind of access used by the nodes in the network: contention access period (CAP), where slotted CSMA/CA is adopted, and contention-free period (CFP), where GTS slots are distributed to the nodes (see Figure 8). Since time slots can have different sizes from one cluster to another, we distribute resources in terms of rate, *i.e.*, *r_j_* is the rate allocated to the j-th sensor. Thereafter, we use these rates to derive the time slot allocation. Note that depending on each specific Superframe (SF) configuration, the maximum transmission rate in each cluster changes. Furthermore, we will also take into account the quality of the radio links by means of the Packet Delivery Ratio (PDR), that is, *PDR_j_* is the ratio between correctly received packets and sent packets at the j-th sensor. As previously commented, beacon collision problems can appear in a cluster-tree WSN based on the beacon enabled mode. For that reason, we take into account a beacon collision mechanism. In particular, we consider the time-division approach adopted by the Zigbee specification [9], *i.e.*, time is divided in such a way that the beacon frame of each coordinator is sent during the inactive periods of the rest of coordinators (see Figure 9).

Given the sensor demands in ***M*** = [*M*_1_, . . ., *M_N_* ]*^T^* and the minimum guaranteed allocation in ***m***= [*m*_1_, . . ., *m_N_* ]*^T^* (both in terms of rate), the optimum allocation is computed by solving the following optimization problem using the CDM,

(27) $$\begin{array}{ccc} \underset{\left\{r_{j}\right\}}{\text{max}} & \sum_{j=1}^{N}U_{j}\left(r_{j}\cdot {\mathrm{PDR}}_{j};w_{j},\gamma \right) & \\ s.t. & m_{j}\leq r_{j}\leq M_{j}, & j=1,\ldots ,N\\ & \mathrm{Ar}≼c & \end{array}$$

where the priority values *w_j_* allow us to balance the allocation towards some sensors in the network and *γ* fixes the degree of fairness as discussed in Equation (2). Moreover, the radio link qualities are also considered as far a *r_j_* · *PDR_j_* is the effective data rate assigned to the j-th user. In particular, note that when *U_j_*(*r_j_*) = *w_j_* log(*r_j_* · *PDR_j_*) the resulting allocation is insensitive to *PDR_j_* because *U_j_*(*r_j_* · *PDR_j_*) = *w_j_*[log(*PDR_j_*) + log(*r_j_*)] and thus, proportional fairness does not benefit sensors with bad channel condition. If we fix *γ* > 1 instead, the resulting allocation is balanced towards the bad sensors. Note that in the limit, *i.e.*, with *γ* → ∞, max-min fairness does not admit low effective data rates even if this implies a significant reduction in terms of the network throughput.

Once the optimal allocation is obtained, it has to be mapped onto a time slot assignment. Since the WSN scenario can be assumed quasi-static in most of the real-life applications, it is reasonable to sustain an allocation for a given number of Beacon Intervals (BI), say *nBI*. Therefore, the real-valued number of time slots assigned to the j-th sensor on the *nBI* periods is

(28) $${\mathrm{TS}}_{j}=\frac{r_{j}^{\prime }\cdot \mathrm{nBI}\cdot T_{\mathrm{BI}}}{b_{j}}$$

where *r′_j_* is the rate across the j-th sensor, that is, its own rate plus the rate of all the nodes below it. Furthermore, *T_BI_* is the BI duration and *b_j_* is the packet size in bits at the j-th sensor. Since *TS_j_* in Equation (28) is real-valued, the cluster head has to round the allocation of its nodes. We propose to allocate first ⌊*TS_j_*⌋ time slots to each node and then successively allocate one extra time slot to the sensors with the biggest value of *TS_j_* − ⌊*TS_j_*⌋, where ⌊·⌋ represents the floor function. Note that the higher the *nBI*, the higher the optimality of the time slot allocation. However, if the network is substantially dynamic, we shall fix *nBI* = 1 and implement other rounding strategies that are out the scope of this paper. In any case, we need algorithms that converge fast to the optimal solution if we want to track the changes in the network efficiently. This is achieved with the proposed CDM as shown in the next simulation results.

## 4. Numerical Results

**Figure 10.**
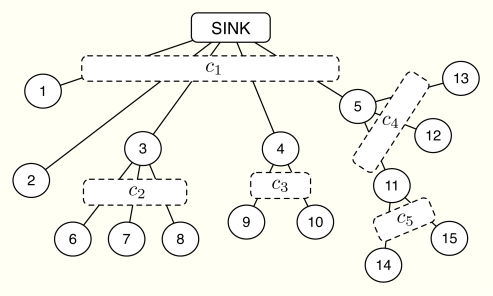
Network configuration used in the numerical results.

Let us consider the network configuration depicted in Figure 10, with 15 sensor nodes, one sink node and 5 capacity constraints that deploy a three-level cluster tree. Note that the following results can be extrapolated to WSNs with a larger number of nodes and a larger number of levels and thus, our simplified example has no loss in generality. Specifically, the Superframe (SF) is configured as follows: *T_BI_* is set to 245.76 ms and is the same at all levels; the SF duration is 61.44 ms at the highest level, 30.72 ms at the intermediate level and 15.36 ms at the lowest level. According to [33], this configuration provides 9.38 kbps, 10.94 kbps and 13.02 kbps of data rate, respectively. Furthermore, if we consider 15 slots per BI dedicated to the Guaranteed Time Slot (GTS) allocation, the size of the slots in bits is 9, 21 and 50, respectively. Accordingly, the rate capacities of the clusters are set to ***c*** = [3.0516, 1.2820, 1.2820, 1.2820, 0.5496]*^T^* kbps.

**Figure 11.**
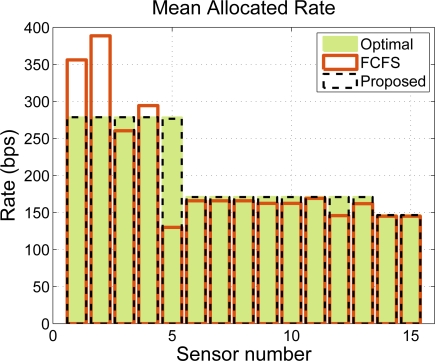
Allocation example for *n* = 20 bits.

In our simulations, we compare our CDM-based approach to the widely-used First Come First Serve (FCFS) policy. We assume, without loss of generality, that the values of *w_j_* and *P DR_j_* are set to 1 and that all the sensors are of the same type and thus, all of them need to transmit *n* bits per BI. However, the rate requests are quantized by the time slot duration at each sensor so that each node asks for *n̄_i_/T_BI_* kbps, where *n̄_i_* is the nearest multiple (above) of the packet size in bits at the i-th node. Figure 11 shows an allocation example for *n* = 20 bits. We notice that the CDM-based policy provides a rate allocation that is very close to the optimal, whereas the FCFS solution does not guarantee a fair resource allocation, even when the throughput is not penalized. Note that, in the example under consideration, there exists no trade-off between fairness and throughput because we have set error-free links. Note also that our proposed time slot allocation does not provide the optimum fair rate allocation due to the rounding effects.

**Figure 12.**
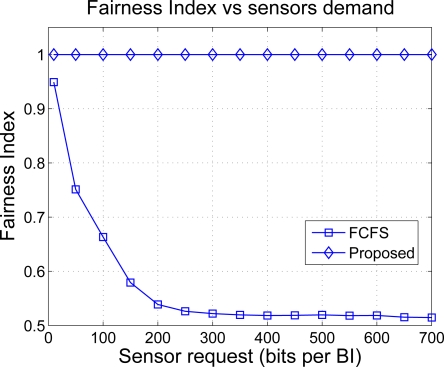
Fairness comparison.

In order to study the fairness degree provided by both solutions, we plot in Figure 12 the Fairness Index (FI) as a function of the requested bits *n*. The FI takes values between 0 and 1 and measures how far is a certain resource allocation with respect to the one that is considered optimal or most fair. If *FI* = 0 the allocation is totally unfair and if *FI* = 1, the resulting allocation is completely fair. A value that is in-between such as 0.6 may be interpreted as follows: the 60% of allocations are considered fair in mean. The FI is computed as [34]

(29) $$\mathrm{FI}=\frac{\sum_{j}z_{j}^{2}}{N{\left(\sum_{j}z_{j}\right)}^{2}}$$

where $z_{j}=\frac{x_{j}}{r_{j}^{*}}$, being $r_{j}^{*}$ the optimal fair allocation and *x_j_* the allocation under test. Therefore, *x_j_* represents the rate allocation that results from either our CDM-based time slot distribution or the standard-proposed FCFS approach. Furthermore, $r_{j}^{*}$ is the real-valued optimal rate allocation, that is, without rounding. Note in the figure that the CDM-based approach provides a solution that is very close to the optimal whereas the FCFS allocation tends to be more unfair as the transmission requirements of the sensors grow until it stabilizes around 0.5, which is pretty unfair. Therefore, FCFS is an acceptable strategy for small traffic loads, that is, when the network resources suffice to satisfy all the sensor requirements. However, the allocation is unfair at high loads because the mechanism does not distinguish sensors that are parents of a sub-cluster tree from sensors that are not. Since the two receive the same amount of resources in mean, the sensors in the sub-cluster tree (including the parent node) are thus penalized. In our example, the routing policy of a sensor is to first relay the packets with a lower time stamp and then send its own packets, which explains the higher allocation of sensors 1 and 2 in Figure 11.

**Figure 13.**
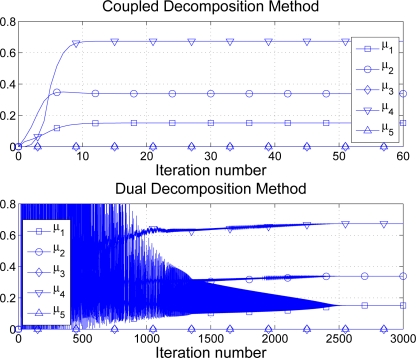
Comparison between the CDM and the dual decomposition.

Finally, signalling is a very important aspect to take into account when considering distributed optimization techniques. The advantage is clear: the network operates at the desired optimal point (proportional fairness in our examples). Notwithstanding, as opposite to other policies such as FCFS, sensors need to exchange information thus draining the sensor batteries. Therefore, distributed optimization approaches make sense if the amount of signalling information is kept small. In Figure 13 we compare the proposed CDM against the classical dual decomposition approach in terms of iterations required to converge to the optimal solution. In this example, requests, maximum transmission rates, minimum guaranteed rates and sensor priorities are picked at random using uniform distributions. In particular, *d_i_*, *c_i_* ∼ 𝒰[0, 50] kbps, *m_i_* ∼ 𝒰[0, 0.5] kbps and *p_i_* ∼ 𝒰[0, 2]. In our simulations, the step-size in dual decomposition is adjusted to provide good performance in general, fixing it to $\alpha^{k}=\frac{0.5}{\sqrt{k}}$ (note that the CDM has no parameter to configure). See in Figure 13 one numerical example. Note that although one iteration of the CDM implies twice the amount of signalling required in one iteration of dual decomposition, the number of iterations in the latter is excessively large and makes the technique impractical in the WSN context. On the contrary, the CDM finds the optimal solution using 10–30 iterations in general. In other words, the CDM requires about 40 *N* to 120 *N* messages to achieve a fair distribution of the available rate whereas the so-called dual decomposition solution needs to exchange up to 5, 000 *N* in our example. If we consider 32-bit messages, our solution requires 56.25 kb in total whereas the dual decomposition approach needs 2343.75 kb of signalling.

## 5. Conclusions

In this paper, we present a resource allocation technique that is designed to fairly distribute the available time slots among the sensors of a cluster-tree WSN. Our solution is based in convex decomposition and, in particular, it combines the so-called primal and dual decompositions in a single algorithm that provides superior performance in terms of convergence speed. Thanks to the proposed Network Utility Maximization (NUM) formulation, it is possible to choose the most adequate definition of fairness in our application, going from max-min fairness to max-sum rate. As shown in the document, the standard-proposed First Come First Serve policy provides pretty unfair allocations when the traffic load in the network increases and proportional fairness is considered. Moreover, the NUM formulation takes into account the different rate capacities in the network, which are due to different superframe configurations, as well as the radio link qualities by means of the Packet Delivery Ratio.

Throughout the document, we focus our attention on the practical application of the proposed method. On one hand, the conversion form rate (required by the algorithm) to time slots is addressed. On the other hand, the signalling involved in the computation of the global optimal solution is detailed. Specifically, our method requires the double amount of signalling per iteration when compared to dual decomposition. Notwithstanding, the number of iterations can be reduced up to a factor of 1,000 so that the total amount of signalling is reduced about 500 times. Besides, the CDM has no parameter to be adjusted, which is a true impairment in practice.

In summary, we can conclude that the most remarkable benefits of our solution are:

- The network can be adjusted to operate with several fairness definitions by fixing the value of *γ*.
- The optimal solution is distributedly computed in the cluster-tree network.
- The price to pay to compute that optimal solution in terms of signalling is minimized with respect to existing techniques.

## Appendix

### Proof of Proposition 3

As it is discussed after Problem (21), the step from ***y****^k^* to ***ŷ****^k^* forces all the values in each subset ${𝒜}_{m}^{k}$ to either increase or decrease their allocation in order to fulfill the corresponding rate constraint. Let us consider the first case, *i.e.*, all the values of ${\hat{y}}_{j}^{k}$ with $j\in {𝒜}_{m}^{k}$ satisfy ${\hat{y}}_{j}^{k}<y_{j}^{k}$. Since $y_{j}^{k}\in [m_{j},M_{j}]$, it is straightforward to see that ${\hat{y}}_{j}^{k}<d_{j}$. However, it is possible that ${\hat{y}}_{j}^{k}\leq m_{j}\forall_{j}\in {𝒜}_{m}^{k}$. Notwithstanding, this implies that there is no strictly feasible point in the original problem, which contradicts the initial hypothesis. Note that we need to increase all the values in ${\hat{y}}_{j}^{k}$ to reach a strictly feasible point but this violates the rate constraint. Therefore, there is at least one value that accomplishes ${\hat{y}}_{j}^{k}\in (m_{j},M_{j})$.

Similarly, we can consider the second case, that is, the values of ${\hat{y}}_{j}^{k}$ with $j\in {𝒜}_{m}^{k}$ are increased in the primal projection. In this occasion, if the resulting point ${\hat{y}}_{j}^{k}$ verifies ${\hat{y}}_{j}^{k}\geq d_{j}\forall_{j}\in {𝒜}_{m}^{k}$, it implies that the associated constraint is not really necessary. However, this contradicts our hypothesis that the subset of active constraints at the optimum is known. This concludes the proof of the proposition.

## References

[1] IEEE (2006). Wireless Medium Access Control (MAC) and Physical Layer (PHY) Specifications for Low-Rate Wireless Personal Area Networks (LR-WPANs).

[2] Koubaa A, Alves M, Tovar E (2005). IEEE 802.15.4 for Wireless Sensor Networks: A Technical Overview.

[3] Chen F, German R, Dressler F Towards IEEE 802.15.4e: A Study of Performance Aspects.

[4] Kim A, Hekland F, Petersen S, Doyle P When HART Goes Wireless: Understanding and Implementing the WirelessHART Standard.

[5] ISA (2009). Wireless Systems for Industrial Automation: Process Control and Related Applications.

[6] Mishra A, Chewoo N, Rosenburgh D On Scheduling Guaranteed Time Slots for Time Sensitive Transactions in IEEE 802.15.4 Networks.

[7] Chitnis M, Pagano P, Lipari G, Liang Y A Survey on Bandwidth Resource Allocation and Scheduling in Wireless Sensor Networks.

[8] IEEE 802.15 WPAN™ Task Group 4b. http://grouper.ieee.org/groups/802/15/pub/TG4b.html.

[9] Zigbee-Alliance ZigBee Specification. http://www.zigbee.org.

[10] Koubaa A, Alves M, Attia M, Van Nieuwenhuyse A Collision-Free Beacon Scheduling Mechanisms for IEEE 802.15.4/Zigbee Cluster-Tree Wireless Sensor Networks.

[11] Morell A, Seco-Granados G, Vicario J (2009). Fair Adaptive Bandwidth and Subchannel Allocation in the WiMAX Uplink. EURASIP J Wireless Communications and Networking.

[12] Morell A (2008). A Convex Decomposition Perspective on Dynamic Bandwidth Allocation and Applications.

[13] The ROLL Design Team (2010). RPL: Routing Protocol for Low Power and Lossy Networks (work in progress). http://tools.ietf.org/html/draft-ietf-roll-rpl-17.

[14] Gnawali O, Fonseca R, Jamieson K, Moss D, Levis P The Collection Tree Protocol (CTP).

[15] Vilajosana X, Llosa J, Pacho J, Vilajosana I, Juan AA, Vicario J, Morell A (2010). ZERO: Probabilistic Routing for Deploy and Forget Wireless Sensor Networks. Sensors.

[16] Notation: ≼, *≺*,≽ and *≺* stand for component-wise inequalities.

[17] Bertsekas D (1999). Nonlinear Programming.

[18] Boyd L, Vandenberghe S (2004). Convex Optimization.

[19] Kelly F (1997). Charging and Rate Control for Elastic Traffic. Eur. Trans. Telecommun.

[20] Kelly F, Maulloo A, Tan D (1998). Rate Control for Communication Networks: Shadow Prices, Proportional Fairness and Stability. J. Oper. Res. Soc.

[21] Mo J, Walrand J (2000). Fair End-to-End Window-based Congestion Control. IEEE/ACM Trans. Netw.

[22] Palomar D, Chiang M (2007). Alternative Decompositions for Distributed Maximization of Network Utility: Framework and Applications. IEEE Trans. Autom. Control.

[23] Xiao L, Johansson M, Boyd S (2004). Simulatenous Routing and Resource Allocation via Dual Decomposition. IEEE Trans. Commun.

[24] Lee J, Chiang M, Calderbank A Network Utility Maximization and Price-Based Distributed Algorithms for Rate-Reliability Tradeoff.

[25] Tseng P, Bertsekas D (1993). On the Convergence of the Exponential Multipliers Method for Convex Programming. Math. Program.

[26] Polyak R (2008). Primal-Dual Exterior Point Method for Convex Optimization. Optim. Method. Softw.

[27] Holmberg K (1995). Primal and Dual Decomposition as Organizational Design: Price and/or Resource Directive Decomposition. Design Models for Hierarchical Organizations: Computation, Information, and Decentralization.

[28] Nedic A, Ozdaglar A (2009). Subgradient Methods for Saddle-Point Problems. JOTA.

[29] The vector ***s*** is a subgradient of the function ***f***: ℝ*^n^* → ℝ at ***x*** ∈ ℝ*^n^* if ***f***(***y***) ≥ ***f***(***x***) + (***y*** − ***x***)*^T^****s*,** ∀***y*** ∈ ℝ*^n^*. If ***f*** is differentiable at ***x***, the subgradient ***s*** and the gradient **∇*f***(***x***) coincide. Otherwise, there exist many subgradients.

[30] Notation: ***δ****_j_* is a zero-valued column vector with its j-th entry equal to 1.

[31] Note that in the case [***A***]_2_***ŷ*** = *c*_2_, Problem (14) is splitted into 2 independent projections: (i) distribute *c*_2_ between sensors 3 and 4 and (ii) distribute *c*_1_ − *c*_2_ between sensors 1 and 2.

[32] We say that a constraint is active when it is attained with equality.

[33] Koubaa A, Alves M, Tovar E GTS Allocation Analysis in IEEE 802.15.4 for Real-Time Wireless Sensor Networks.

[34] Jain R, Chiu D, Hawe W (1984). A Quantitative Measure of Fairness and Discrimination for Resource Allocation in Shared Systems.

